# *VacA*, *cagA*, *iceA* and *oipA* genotypes status and antimicrobial resistance properties of *Helicobacter pylori* isolated from various types of ready to eat foods

**DOI:** 10.1186/s12941-015-0115-z

**Published:** 2016-01-20

**Authors:** Behsan Hemmatinezhad, Hassan Momtaz, Ebrahim Rahimi

**Affiliations:** Graduated Student of Veterinary Medicine, College of Veterinary Medicine, Islamic Azad University, Shahrekord, Iran; Department of Microbiology, College of Basic Sciences, Islamic Azad University, Shahrekord, Iran; Department of Food Hygiene and Public Health, College of Veterinary Medicine, Islamic Azad University, Shahrekord, Iran

**Keywords:** *Helicobacter pylori*, Genotypes, Genotyping, Ready to eat foods

## Abstract

**Background:**

Despite the high clinical standing of *Helicobacter pylori*, its exact routes of transmission and origin have not been determined. Based on the contentious hypothesis, foods play an important roles in the transmission of *H. pylori* to humans. The present study was carried out to investigate the *vacA*, *cagA*, *oipA* and *iceA* genotypes status of *H. pylori* isolated from the various types of ready to eat foods.

**Methods:**

A total of 550 ready to eat food samples were cultured and tested. *H. pylori*-positive strains were analyzed for the presence of various genotypes and antimicrobial resistance pattern.

**Results:**

Seventy four out of 550 (13.45 %) samples were positive for *H. pylori*. Olvie salad (36 %), restaurant salad (30 %), fruit salad (28 %) and soup (22 %) were the most commonly contaminated. *H. pylori* strains harbored the highest levels of resistance against amoxicillin (94.59 %), ampicillin (93.24 %), metronidazole (89.18 %) and tetracycline (72.97 %). The most commonly detected genotypes were *vacA s1a* (78.37 %), *vacA m2* (75.67 %), *vacA m1a* (51.35 %) and *cagA* (41.89 %). The prevalence of *iceA1*, *iceA2* and *oipA* genotypes were 13.51, 4.05 and 18.91 %, respectively. *S1am2* (70.27 %), *s1am1a* (39.18 %) and *m1am2* (31.08 %) were the most commonly detected combined genotypes. Of 40 different genotypic combinations, s1a/cagA+/iceA1/oipA− (12.16 %), s1a/cagA+/iceA1/oipA+ (10.81 %) and s1a/cagA−/iceA1/oipA+ (10.81 %) were the most prevalent.

**Conclusions:**

The present investigation showed that some types of ready to eat food samples maybe the sources of resistant and virulent strains of *H. pylori*. Warily use of antibiotics with respect to the results of disk diffusion method and careful health monitoring on food and staffs of food producing companies maybe reduce the risk of *H. pylori* in foods.

## Background

Ready to eat foods play an important roles in the nutrition of Iranian people. Every day millions of people use from ready to eat foods in their main meals. Therefore, hygienic quality of ready to eat foods is extremely important regarding public health hazards.

*Helicobacter pylori* (*H. pylori*) is a microaerophilic gram-negative bacterium which is known as the causative agent of peptic ulcer disease, type B gastritis, gastric adenocarcinoma and mucosa-associated lymphoid tissue lymphoma [1]. It has been estimated that 17–86 % of hospitalized patients with peptic ulcers were infected with *H. pylori* [1–3].

The role of foods in the transmission of *H. pylori* is still unknown but there were several investigations which focused on the identification of this bacterium in various types of food samples [4–8]. Good conditions for the growth of bacteria in various types of foods cause it to be survive [6, 8].

To appraise the pathogenicity of *H. pylori*, evaluation of latent virulence factors and genotypes is essential. The most commonly important virulence factors among *H. pylori* strains of different clinical outcomes of human and animal beings are the vacuolating cytotoxin (*vacA*), induced by contact with the epithelium antigen (*iceA*), cytotoxin associated gene (*cag*) and outer inflammatory protein (*oip*) [6, 9, 10]. These genes are usually induced adhesion and invasion to the gastric epithelial cells [11, 12]. The *vacA* belongs to the group of genes with variable genotypes or structures. This gene is associated with injury to epithelial cells. The *vacA* gene is polymorphic, comprising variable signal regions (type *s1* or *s2*) and mid-regions (type *m1* or *m2*). The *s1* type is further subtyped into *s1a*, *s1b* and *s1c* subtypes and the *m1* into *m1a* and *m1b* subtypes. The mosaic combination of s and m-region allelic types determines the particular cytotoxin and consequently, the pathogenicity of the bacterium [11, 13]. The *iceA* gene has two main allelic variants *iceA1* and *iceA2* but their functions are not yet clear. *Cag* pathogenicity island (PAI) has been shown to be involved in persuading inflammation, ulceration and carcinogenesis [12]. The *cagA* gene has been detected in the specimens taken from the severe cases of peptic ulcer [8–12]. The *oipA* gene plays a significant role in effective colonization of mucosa [8–12]. Genotyping using these virulence markers is considered as one of the best approaches for study of correlations between *H*. *pylori* isolates from different samples.

Data on the epidemiology and transmission of *H. pylori* is extremely significant in order to prevent its distribution and to identify high-risk populations, especially in areas that have high rates of gastritis, peptic ulcers and gastric cancer such as Iran [6–10, 13]. Considering the unclear epidemiological aspects of *H. pylori* in ready to eat foods and according to the high prevalence of *H*. *pylori* all-around the world, the present investigation was carried out in order to study the exact status of *vacA*, *cagA*, *iceA* and *oipA* genotypes and antibiotic resistance pattern of *H. pylori* isolated from various types of ready to eat food samples.

## Methods

### Sample collection

From December 2013 to May 2014, overall 550 ready to eat food samples including cream-candy (n = 50), traditional bread (n = 50), sausage (n = 50), salami (n = 50), hamburger (n = 50), soup (n = 50), restaurant salad (n = 50), falafel (n = 50), olvie salad (n = 50), chicken nugget (n = 50) and fruit salad (n = 50) were purchased from the supermarkets of Isfahan province, Iran. Samples (100 mL, in sterile glass containers) were transported to the laboratory at 4 °C. All samples were kept under refrigeration in plastic bags; information about dates of production and of assigned shelf lives was not presented.

### Isolation of *Helicobacter pylori*

Twenty five milliliters of each homogenized sample were added to 225 mL of Wilkins Chalgren anaerobe broth (Oxoid, UK) supplemented with colistin methanesulfonate (30 mg/L) and 5 % of horse serum (Sigma, St. Louis, MO, USA) and nalidixic acid (30 mg/L), vancomycin (10 mg/L) cycloheximide (100 mg/L) and trimethoprim (30 mg/L) (Sigma, St. Louis, MO, USA) and incubated for 7 days at 37 °C with shaking under microaerophilic conditions. Then, 0.1 mL of the enrichment selective broth was plated onto Wilkins Chalgren anaerobe agar (Oxoid, UK) supplemented with 5 % of defibrinated horse blood and 30 mg/L colistin methanesulfonate, 100 mg/L cycloheximide, 30 mg/L nalidixic acid, 30 mg/L trimethoprim, and 10 mg/L vancomycin (Sigma, St. Louis, MO, USA) and incubated for 7 days at 37 °C under microaerophilic conditions. For comparison, a reference strain of *H. pylori* (ATCC 43504) was employed.

### DNA extraction and *Helicobacter pylori* 16S rRNA gene amplification

Suspected colonies were identified as *H. pylori* based on the PCR technique. Genomic DNA was extracted from the colonies with typical characters of *H. pylori* using a DNA extraction kit for cells and tissues (Roche Applied Science, Germany, 11814770001) according to the manufacturer’s instructions and its density was assessed by optic densitometry. Extracted DNA was amplified for the *16S rRNA* gene (primers: HP-F: 5′-CTGGAGAGACTAAGCCCTCC-3′ and HP-R: 5′-ATTACTGACGCTGATTGTGC-3′) [14]. PCR reactions were performed in a final volume of 50 µL containing 5 µL 10 × buffer + MgCl_2_, 2 mM dNTP, 2 unit Taq DNA polymerase, 100 ng genomic DNA as a template, and 25 pmol of each primer. PCR was performed using a thermal cycler (Eppendorf Co., Germany) under the following conditions: an initial denaturation for 2 min at 94 °C; 30 cycles of 95 °C for 30 s, 60 °C for 30 s, and 72 °C for 30 s and a final extension at 72 °C for 8 min.

### Antimicrobial susceptibility testing

Pure cultures of *H. pylori* were applied for antibiotic susceptibility test. One strain from each *H. pylori*-positive sample was selected for this aim. Antimicrobial susceptibility test was accomplished by the Kirby-Bauer disc diffusion method using Mueller–Hinton agar (Merck, Germany) supplemented with 5 % defibrinated sheep blood and 7 % fetal calf serum, according to the Clinical Laboratory Standards Institute [15]. The antimicrobial resistance of *H. pylori* was measured against the widely used antibiotics in cases of *H. pylori* gastric ulcer. The following antimicrobial disks (HiMedia Laboratories, Mumbai, India) were used: ampicillin (10 µg), metronidazole (5 µg), erythromycin (5 µg), clarithromycin (2 µg), amoxicillin (10 µg), tetracycline (30 µg), levofloxacin (5 µg), streptomycin (10 µg), rifampin (30 µg), cefsulodin (30 µg), trimethoprim (25 µg), furazolidone (1 µg) and spiramycin (100 µg). After incubation at 37 °C for 48 h in a microaerophilic atmosphere, the susceptibility of the *H. pylori* was measured against each antimicrobial agents. Results were construed in accordance with interpretive criteria provided by CLSI (2012) [15]. The *H. pylori* ATCC 43504 was used as quality control organisms in antimicrobial susceptibility determination.

### Genotyping of vacA, cagA, iceA and oipA genes of *Helicobacter pylori*

Presence of the *oipA*, *cagA* and the genotypes of *vacA* (*s1a*, *s1b*, *s1c*, *m1a*, *m1b* and *m2*) and *iceA* (*iceA1* and *iceA2*) alleles were determined using PCR technique. List of primers is shown in Table 1 [12, 16–21].

**Table 1:** Oligonucleotide primers used for genotyping of *Helicobacter pylori* strains isolated from ready to eat food samples

Genes name	Primer Sequence (5′–3′)	Size of product (bp)
*vacA s*_*1*_ *a*	F: CTCTCGCTTTAGTAGGAGC R: CTGCTTGAATGCGCCAAAC	213
*vacA s*_*1*_ *b*	F: AGCGCCATACCGCAAGAG CTGCTTGAATGCGCCAAAC	187
*vacA s*_*1*_ *c*	F: CTCTCGCTTTAGTGGGGYT R: CTGCTTGAATGCGCCAAAC	213
*vacA s*_*2*_	F: GCTAACACGCCAAATGATCC R: CTGCTTGAATGCGCCAAAC	199
*vacA m*_*1*_ *A*	F: GGTCAAAATGCGGTCATGG R: CCATTGGTACCTGTAGAAAC	290
*vacA m*_*1*_ *B*	F: GGCCCCAATGCAGTCATGGA R: GCTGTTAGTGCCTAAAGAAGCAT	291
*vacA m*_*2*_	F: GGAGCCCCAGGAAACATTG R: CATAACTAGCGCCTTGCA	352
*cag A*	F: GATAACAGCCAAGCTTTTGAGG R: CTGCAAAAGATTGTTTGGCAGA	300
*iceA*_*1*_	F: GTGTTTTTAACCAAAGTATC R: CTATAGCCASTYTCTTTGCA	247
*iceA*_*2*_	F: GTTGGGTATATCACAATTTAT R: TTRCCCTATTTTCTAGTAGGT	229/334
*oip A*	F: GTTTTTGATGCATGGGATTT R: GTGCATCTCTTATGGCTTT	401

The PCR were performed in a total volume of 50 μl containing 1 μM of each primers, 1 μL of genomic DNA (approximately 200 ng), 1 mM of dNTPs mix (invitrogen), 2 mM of Mgcl2, and 0.05 U/μL Taq DNA polymerase (invitrogen). PCR amplifications were performed in an automated thermal cycler (Biometra Co., Germany). The following cycle conditions were used for PCR amplification: for *vacA*: 32 cycles of 45 s at 95 °C, 50 s at 64 °C, and 70 s at 72 °C; for *cagA*: 1 min at 94 °C, 1 min at 56 °C, and 1 min at 72 °C; for *iceA*: 1 min at 94 °C, 1 min at 56 °C, and 1 min at 72 °C and finally, for *oipA*: 1 min at 94 °C, 1 min at 56 °C and 1 min at 72 °C. All runs included one negative DNA control consisting of PCR grade water and two or more positive controls (26695, J99, SS1, Tx30, 88–23 and 84–183).

### Gel electrophoresis

The PCR amplification products (10 μl) were subjected to electrophoresis in a 1.5 % agarose gel in 1X TBE buffer at 80 V for 30 min, stained with ethidium bromide, and images were obtained in a UVIdoc gel documentation systems (UK). The PCR products were identified by 100 bp DNA size marker (Fermentas, Germany).

### Statistical analysis

Data were transferred to Microsoft Excel spreadsheet (Microsoft Corp., Redmond, WA, USA) for analysis. Using SPSS 16.0 statistical software (SPSS Inc., Chicago, IL, USA), Chi square test and Fisher’s exact two-tailed test analysis was performed and differences were considered significant at values of *P* < 0.05. Distribution of *H. pylori* genotypes isolated from food stuffs were statistically analyzed.

## Results

### Prevalence of *Helicobacter pylori* in various types of ready to eat food samples

All of the ready to eat food samples were examined using the culture and PCR techniques. Table 2 shows the total distribution of *H. pylori* in the ready to eat food samples. Of 550 ready to eat food samples, 74 (13.45 %) were positive for *H. pylori*. The most commonly contaminated samples were olvie salad (36 %), restaurant salad (30 %), fruit salad (28 %) and soup (22 %). There were no positive results for sausage, salami and chicken nugget. There were statistically significant differences amongst the incidence of bacteria in hamburger and olvie salad (*P* = 0.027), traditional bread and restaurant salad (*P* = 0.033) and soup and falafel (*P* = 0.041).

**Table 2:** Distribution *of Helicobacter pylori* in various types of ready to eat food samples

Types of samples	No. samples collected	Positive results for *H. pylori* (%)
Cream-candy	50	9 (18)
Traditional bread	50	3 (6)
Sausage	50	–
Salami	50	–
Hamburger	50	1 (2)
Soup	50	11 (22)
Restaurant salad	50	15 (30)
Falafel	50	3 (6)
Olvie salad	50	18 (36)
Chicken nugget	50	–
Fruit salad	50	14 (28)
Total	550	74 (13.45)

### Antimicrobial susceptibility pattern

Antimicrobial susceptibility of *H. pylori* isolates of ready to eat food samples is shown in Table 3. *H. pylori* strains showed the highest levels of resistance against amoxicillin (94.59 %), ampicillin (93.24 %), metronidazole (89.18 %) and tetracycline (72.97 %) antibiotics. There were significant difference between the levels of antibiotic resistance and sources of *H. pylori* strains (*P* = 0.039). There were statistically significant differences in the levels of antibiotic resistance between amoxicillin and spiramycin (*P* = 0.015), ampicillin and spiramycin (*P* = 0.018), metronidazole and furazolidone (*P* = 0.023), tetracycline and furazolidone (*P* = 0.027), amoxicillin and furazolidone (*P* = 0.025), metronidazole and rifampin (*P* = 0.031), ampicillin and erythromycin (*P* = 0.026) and amoxicillin and cefsulodin (*P* = 0.022).

**Table 3:** Antimicrobial resistance pattern of *Helicobacter pylori* isolates of ready to eat food samples

Types of samples (no. positive results)	Pattern of antibiotic resistance (%)												
	AM10	Met5	ER5	CLR2	AMX 10	Tet30	Lev5	S10	RIF30	Cef30	TRP25	FZL1	Spi100
Cream-candy (9)	8 (88.88)	9 (100)	6 (66.66)	4 (44.44)	8 (88.88)	7 (77.77)	3 (33.33)	3 (33.33)	2 ()	3 (33.33)	5 (55.55)	1 (11.11)	1 (11.11)
Traditional bread (3)	3 (100)	3 (100)	2 (66.66)	1 (33.33)	3 (100)	2 (66.66)	1 (33.33)	1 (33.33)	1 (33.33)	1 (33.33)	1 (33.33)	–	–
Hamburger (1)	1 (100)	–	1 (100)	–	1 (100)	1 (100)	–	1 (100)	–	–	1 (100)	–	–
Soup (11)	10 (90.90)	10 (90.90)	6 (54.54)	5 (45.45)	10 (90.90)	8 (72.72)	4 (36.36)	3 (27.27)	3 (27.27)	3 (27.27)	6 (54.54)	2 (18.18)	1 (9.09)
Restaurant salad (15)	13 (86.66)	13 (86.66)	8 (53.33)	6 (40)	14 (93.33)	10 (66.66)	5 (33.33)	4 (26.66)	4 (26.66)	4 (26.66)	7 (46.66)	4 (26.66)	2 (13.33)
Falafel (3)	3 (100)	3 (100)	2 (66.66)	1 (33.33)	3 (100)	2 (66.66)	2 (66.66)	1 (33.33)	1 (33.33)	1 (33.33)	2 (66.66)	1 (33.33)	–
Olvie salad (18)	16 (88.88)	16 (88.88)	10 (55.55)	9 (50)	17 (94.44)	13 (72.22)	6 (33.33)	5 (27.77)	5 (27.77)	6 (33.33)	10 (55.55)	6 (33.33)	3 (16.66)
Fruit salad (14)	13 (92.85)	12 (85.71)	8 (57.14)	11 (78.57)	14 (100)	11 (78.57)	6 (42.85)	5 (35.71)	5 (35.71)	4 (28.57)	8 (57.14)	5 (35.71)	2 (14.28)
Total (74)	69 (93.24)	66 (89.18)	43 (58.10)	34 (45.94)	70 (94.59)	54 (72.97)	27 (36.48)	23 (31.08)	21 (28.37)	22 (29.72)	40 (54.05)	19 (25.67)	9 (12.16)

*AM10* ampicillin (10 µg), *Met5* metronidazole (5 µg), *ER5* erythromycin (5 µg), *CLR2* clarithromycin (2 µg), *AMX10* amoxicillin (10 µg), *Tet30* tetracycline (30 µg), *Lev5* levofloxacin (5 µg), *S10* streptomycin (10 µg), *RIF30* rifampin (30 µg), *Cef30* cefsulodin (30 µg), *TRP25* trimethoprim (25 µg), *FZL1* furazolidone (1 µg) and *Spi100* spiramycin (100 µg)

### Distribution of *Helicobacter pylori* genotypes

Distribution of *vacA*, *cagA*, *iceA* and *oipA* genotypes of the *H. pylori* strains of ready to eat food samples is shown in Table 4. We found that the most commonly detected genotypes were *vacA s1a* (78.37 %), *vacA m2* (75.67 %), *vacA m1a* (51.35 %) and *cagA* (41.89 %). Total prevalence of *iceA1*, *iceA2* and *oipA* genotypes were 13.51, 4.05 and 18.91 %, respectively. Significant differences were found between the incidence of *s1a* and *s1c* (*P* = 0.028) and also between *s1a* and *s2* (*P* = 0.021), *s1a* and *m1b* (*P* = 0.019), *m2* and *s1c* (*P* = 0.023), *m1a* and *m1b* (*P* = 0.024), *m2* and *s2* (*P* = 0.030), *m2* and *m1b* (*P* = 0.036), *iceA1* and *iceA2* (*P* = 0.042) and *cagA* and *oipA* (*P* = 0.037) genotypes. Fifteen different genotypic combinations were detected in the *H. pylori* isolates of ready to eat food samples (Table 5). The most commonly detected combined genotypes were *s1am2* (70.27 %), *s1am1a* (39.18 %) and *m1am2* (31.08 %). There were no positive results for *m1bs2* genotype. Significant differences were found between the incidence of *s1am2* and *s1am1b* (*P* = 0.025), *s1am2* and *s1cm1b* (*P* = 0.039), *s1am1a* and *s2m2* (*P* = 0.041), *s1am2* and *m1bm2* (*P* = 0.043), *m2s1a* and *iceA1iceA2* (*P* = 0.025).

**Table 4:** Distribution of various genotypes in *Helicobacter pylori* strains of ready to eat food samples

Types of samples (no. positive results)	Distribution of *vacA, cagA, iceA and oipA* genotypes (%)										
	*S1a*	*S1b*	*S1c*	*S2*	*M1a*	*M1b*	*M2*	*CagA*	*IceA1*	*IceA2*	*OipA*
Cream-candy (9)	7 (77.77)	3 (33.33)	1 (11.11)	–	4 (44.44)	2 (22.22)	7 (77.77)	1 (11.11)	2 (22.22)	–	3 (33.33)
Traditional bread (3)	2 (66.66)	–	–	–	1 (33.33)	–	2 (66.66)	1 (33.33)	–	–	–
Hamburger (1)	1 (100)	–	–	–	–	–	–	1 (100)	–	–	–
Soup (11)	11 (100)	4 (36.36)	2 (18.18)	1 (9.09)	4 (36.36)	3 (27.27)	10 (90.90)	9 (81.81)	3 (27.27)	1 (9.09)	4 (36.36)
Restaurant salad (15)	10 (66.66)	4 (26.66)	3 (20)	2 (13.33)	8 (53.33)	1 (6.66)	11 (73.33)	2 (13.33)	2 (13.33)	1 (6.66)	3 (20)
Falafel (3)	2 (66.66)	1 (33.33)	–	–	1 (33.33)	–	1 (33.33)	1 (33.33)	–	–	–
Olvie salad (18)	16 (88.88)	5 (27.77)	4 (22.22)	2 (11.11)	13 (72.22)	2 (11.11)	15 (83.33)	15 (83.33)	2 (11.11)	1 (5.55)	2 (11.11)
Fruit salad (14)	9 (64.28)	3 (21.42)	3 (21.42)	1 (7.14)	7 (50)	–	10 (71.42)	1 (7.14)	1 (7.14)	–	2 (14.28)
Total (74)	58 (78.37)	20 (27.02)	13 (17.56)	6 (8.10)	38 (51.35)	8 (10.81)	56 (75.67)	31 (41.89)	10 (13.51)	3 (4.05)	14 (18.91)

**Table 5:** Distribution of combined genotypes of *Helicobacter pylori* isolated from ready to eat foods

Genotypes	Prevalence (%)^a^
M1as1a	29 (39.18)
M1as1b	12 (16.21)
M1bs1a	4 (5.40)
M1bs1b	2 (2.70)
M1as1c	3 (4.05)
M1bs1c	1 (1.35)
M2s1a	52 (70.27)
M2s1b	11 (14.86)
M2s1c	6 (8.10)
M2s2	1 (1.35)
M1as2	1 (1.35)
M1bs2	–
M1am2	23 (31.08)
M1bm2	2 (2.70)
CagA+	31 (41.89)
CagA−	43 (58.10)
IceA1	10 (13.51)
IceA2	3 (4.05)
IceA1 IceA2	1 (1.35)
OipA+	14 (18.91)
OipA−	60 (81.08)

^a^From a total of 74 positive strains of *H. pylori*

### Distribution of combined genotypes

Forty different genotypic combinations were detected in the *H. pylori* strains of food stuffs (Table 6). The most commonly detected combined genotypes were s1a/cagA+/iceA1/oipA− (12.16 %), s1a/cagA+/iceA1/oipA+ (10.81 %), s1a/cagA−/iceA1/oipA+ (10.81 %), s1b/cagA+/iceA1/oipA− (9.45 %), m2/cagA+/iceA1/oipA+ (9.45 %), m2/cagA+/iceA1/oipA− (9.45 %), m2/cagA−/iceA1/oipA+ (9.45 %) and m2/cagA−/iceA1/oipA− (9.45 %).

**Table 6:** Combined *vacA*, *cagA*, *iceA* and *oipA* genotypes of *Helicobacter pylori* isolated from ready to eat food samples

Combined genotypes	Total distribution (%)^a^
s1a/cagA+/iceA1/oipA+	8 (10.81)
s1b/cagA+/iceA1/oipA+	5 (6.75)
s1c/cagA+/iceA1/oipA+	2 (2.70)
s1a/cagA+/iceA2/oipA+	1 (1.35)
s1b/cagA+/iceA2/oipA+	–
s1c/cagA+/iceA2/oipA+	–
s1a/cagA−/iceA1/oipA+	8 (10.81)
s1b/cagA−/iceA1/oipA+	6 (8.10)
s1c/cagA−/iceA1/oipA+	2 (2.70)
s1a/cagA−/iceA2/oipA+	1 (1.35)
s1b/cagA−/iceA2/oipA+	–
s1c/cagA−/iceA2/oipA+	–
s1a/cagA+/iceA1/oipA−	9 (12.16)
s1b/cagA+/iceA1/oipA−	7 (9.45)
s1c/cagA+/iceA1/oipA−	3 (4.05)
s2/cagA+/iceA1/oipA+	3 (4.05)
s2/cagA+/iceA2/oipA+	–
s2/cagA−/iceA1/oipA+	4 (5.40)
s2/cagA−/iceA2/oipA+	–
s2/cagA−/iceA2/oipA−	–
s2/cagA+/iceA2/oipA−	1 (1.35)
s2/cagA+/iceA1/oipA−	2 (2.70)
m1a/cagA+/iceA1/oipA+	5 (6.75)
m1b/cagA+/iceA1/oipA+	2 (2.70)
m1a/cagA+/iceA2/oipA+	1 (1.35)
m1b/cagA+/iceA2/oipA+	–
m1a/cagA−/iceA1/oipA+	6 (8.10)
m1b/cagA−/iceA1/oipA+	3 (4.05)
m1a/cagA−/iceA2/oipA+	–
m1b/cagA−/iceA2/oipA+	–
m1a/cagA+/iceA1/oipA−	5 (6.75)
m1b/cagA+/iceA2/oipA−	2 (2.70)
m2/cagA+/iceA1/oipA+	7 (9.45)
m2/cagA+/iceA2/oipA+	1 (1.35)
m2/cagA+/iceA2/oipA−	1 (1.35)
m2/cagA+/iceA1/oipA−	7 (9.45)
m2/cagA−/iceA1/oipA+	7 (9.45)
m2/cagA−/iceA2/oipA+	1 (1.35)
m2/cagA−/iceA2/oipA−	1 (1.35)
m2/cagA−/iceA1/oipA−	7 (9.45)

^a^From a total of 74 positive strains of *H. pylori*

## Discussion

The results of our study showed that 13.45 % of ready to eat food samples were contaminated with *H. pylori* which was entirely high. In comparison with other investigations which were conducted on food staffs, the prevalence of *H. pylori* in our study was higher than those of milk (12.5 %) [22] and vegetable and salad (10.86 %) [7] but was lower than dairy products (19.2 %) [6], vegetable (13.68 %) [8] and restaurant salad (14 %) [8]. To date, various studies have been conducted on the prevalence of *H. pylori* in foods with animal origin [22–25]. In a study accompanied in Italy [23], the *H. pylori* was detected in more than 25 % of foods with animal origin. Japanese researchers reported the higher levels of contamination in foods with animal origin (72.2 %) [26]. In despite of other foods with animal origin [22–26], prevalence of *H. pylori* in the hamburger, olvie salad and soup (meat based ready to eat food stuffs) of our study were 2, 36 and 22 %, respectively. Prevalence of bacterium in Greek [24] and USA [25] were 20 % and 60 %, respectively which was higher than our results.

One of the most important substances in the preparation of sausage, salami, hamburger, olvie salad, chicken nugget and soup is meat. There were no positive results for chicken nugget, sausage and salami samples of our study. It is maybe related to the application of high temperature during processing of these products. In addition, observation of hygienic conditions in the preparation and packaging of these products maybe another reason for lack of *H. pylori*. In comparison with sausage, salami and chicken nugget samples, the *H. pylori* had significant prevalence in hamburger (2 %), olvie salad (36 %) and soup (22 %) samples. High amount of water activity (Aw) in these food samples, optimum pH and salt levels, presence of appropriate levels of amino acids including arginine, histidine, isoleucine, leucine, methionine, phenylalanine, alanine, valine, proline, serine, and tryptophan which are necessary for growth of *H. pylori* [27], presence of vegetables in some of these foods (olvie salad and soup) which are maybe reservoir for *H. pylori* [7, 8], unsanitary conditions in their preparation and finally cross contamination of these foods due to handling by factory and food units staffs are the main reasons for the high prevalence of *H. pylori* in these samples. The results of Mhaskar et al. [28] confirmed the finding of our study about the risk of meat products for *H. pylori* infection. They reported that the prevalence of peptic ulcer and *H. pylori* infection were entirely higher in those patients which were used from meat and meat products [odds ratio (OR) 2.35, 95 % confident interval (CI): 1.30–4.23] and restaurant foods (OR 3.77, 95 % CI 1.39–10.23) than vegans.

Preparation of some kinds of these food samples including falafel (pea based fast food with some kinds of vegetables), cream-candy, restaurant salad, traditional bread, fruit salad and olvie salad need moderate levels of water. Roles of contaminated water and even drinking water in transmission of *H. pylori* have been confirmed previously [29–32]. Probably, the water sources used for production and processing of these foods samples were contaminated. Finally, using from unsanitary conditions, handling contamination, use of contaminated equipment and lack of public and individual hygiene are the main reasons for the high prevalence of *H. pylori* in food samples of our study. Food safety regulations as well as quality standards—including good agricultural practices (GAPs), good manufacturing practices (GMPs) and hazard analysis and critical control points (HACCP)—should be introduced in Iranian food units and factories in order to control contamination and proliferation of pathogenic bacteria.

Another part of our investigation focused on the distribution of *vacA*, *cagA*, *oipA* and *iceA* genotypes. Results showed that *vacA s1a* (78.37 %), *vacA m2* (75.67 %), *vacA m1a* (51.35 %) and *cagA* (41.89 %) were the most commonly detected genotypes in *H. pylori* strains of ready to eat foods. Our results showed that the *cagA* gene had the highest prevalence in the hamburger (100 %), soup (81.81 %) and olvie salad (83.33 %). As we said, these are a meat based ready to eat food samples and the high prevalence of *cagA* gene in these samples maybe showed the high presence of *cagA* positive strains of *H. pylori* in the meat used for production of these food stuffs. Yahaghi et al. [8] reported that the *oipA* (86.44 %), *cagA* (57.62 %), *vacA s1a* (37.28 %) and *vacA m1a* (30.50 %) were the most commonly detected genotypes in the *H. pylori* isolates of vegetable and salad. They showed that *vacA s1c* (10.16 %) had low prevalence which was similar to our findings. *VacA s1a*, *m2* and *m1a* and *cagA* were the most commonly detected genotypes in the clinical samples of human beings [9, 10, 13, 33], foods [6, 8]) and those of animal sources [9]. Close association of *vacA* and *cagA* genotypes with interleukin 8 (IL-8) production, cytotoxin production, gastric epithelial cells adhesion, inflammatory effects, vacuolization and apoptosis in gastric epithelial cells has been observed previously [34, 35]. Since *H. pylori* isolates in our study harbored *vacA* and *cagA* genotypes, consumption of ready to eat foods contaminated with virulent strains may provoke duodenal ulceration, gastric mucosal atrophy and gastric cancer.

Another important finding of our investigation is about the presence of high antibiotic resistance in the *H. pylori* strains of ready to eat food samples. Our results revealed that the *H. pylori* strains of ready to eat food samples had the high levels of antibiotic resistance against ampicillin, metronidazole, erythromycin, amoxicillin, tetracycline and trimethoprim. Similar findings have been reported previously by Yahaghi et al. [8], Thyagarajan et al. [36], Secka et al. [37] and Bang et al. [38]. Low levels of antibiotic resistance in the *H. pylori* strains against spiramycin, furazolidone, cefsulodin, rifampin, streptomycin and levofloxacin is may be due to the regular and low prescription of these antibiotics. Epidemiological investigations of Nigeria, Senegal, Iran, India, China, Taiwan, Saudi-Arabia, Thailand, Egypt, Brazil, Colombia and Argentina showed that the *H. pylori* isolates of human clinical specimens had the highest levels of resistance against metronidazole, amoxicillin, quinolones and tetracycline [39] which was similar to our results.

The results of antimicrobial resistance pattern had indirectly confirmed that the *H. pylori* strains of hamburger, soup and olvie salad (meat based ready to eat foods) were transferred from the meat samples of infected animals. It is because of the *H. pylori* strains of these food stuffs were relatively resistant to the antibiotics used especially in the veterinary medicine. Prescription of metronidazole, clarithromycin, levofloxacin, rifampin, cefsulodin, furazolidone and spiramycin antimicrobial agents is not routine in the cases of diseases in animals. Therefore, the prevalence of resistance against these antibiotics in the *H. pylori* strains of hamburger, soup and olvie salad samples which are relatively made from meat is low and this finding can indirectly confirm the primary infections of meat used from production of hamburger, soup and olvie salad.

## Conclusions

In conclusion, ready to eat foods in Iran harbor *H. pylori* strains with high prevalence of *vacA*, *cagA*, *iceA* and *oipA* genotypes. High prevalence of *H. pylori* in our samples suggest that contaminated ready to eat foods maybe the sources of the bacteria and that it entered the human population in period of time. Diversity of *H. pylori* genotypes between various types of ready to eat food samples maybe showed that there were various sources of contamination for these food stuffs. Bacterial strains had the lowest levels of resistance against spiramycin and furazolidone which was quite interesting. The most important finding of this study is that the ready to eat food stuffs harbored virulent and resistant strains of *H. pylori*. Carefully prescription of antibiotics with respect to the results of disk diffusion method and cautious health monitoring on food and staffs of food producing companies maybe reduce the risk of virulent and resistant strains of *H. pylori* in Iranian food samples.

## Abbreviations

*H. pylori*: *Helicobacter pylori*
*VacA*: vacuolating cytotoxin
*IceA*: induced by contact with the epithelium antigen
*Cag*: cytotoxin associated gene
*Oip*: outer inflammatory protein
PCR: polymerase chain reaction
